# A mechanophysical phase transition provides a dramatic example of colour polymorphism: the tribochromism of a substituted tri(methylene)tetrahydrofuran-2-one

**DOI:** 10.1186/s13065-014-0070-3

**Published:** 2014-12-16

**Authors:** Abdullah M Asiri, Harry G Heller, David S Hughes, Michael B Hursthouse, John Kendrick, Frank JJ Leusen, Riccardo Montis

**Affiliations:** 1 Center of Excellence for Advanced Materials Research (CEAMR), King Abdulaziz University, Jeddah, 21589 Saudi Arabia; 2 School of Chemistry, Cardiff University, Cardiff, CF10 3AT Wales UK; 3 Chemistry, Faculty of Natural and Life Sciences, University of Southampton, Southampton, SO17 1BJ UK; 4 Department of Chemistry, Faculty of Science, King Abdulaziz University, Jeddah, 21588 Saudi Arabia; 5 School of Life Sciences, University of Bradford, Richmond Road, Bradford, BD7 1DP UK; 6 Dipartimento di Scienze Chimiche e Geologiche, Università degli Studi di Cagliari, Cittadella Universitaria, Monserrato, I-09042 CA Italy

**Keywords:** Photochromism, Tribochromism, Crystal structure analysis, Molecular and lattice energy calculations, Colour polymorphism

## Abstract

**Background:**

Derivatives of fulgides have been shown to have interesting photochromic properties. We have synthesised a number of such derivatives and have found, in some cases, that crystals can be made to change colour on crushing, a phenomenon we have termed “tribochromism”. We have studied a number of derivatives by X-ray crystallography, to see if the colour is linked to molecular structure or crystal packing, or both, and our structural results have been supported by calculation of molecular and lattice energies.

**Results:**

A number of 5-dicyanomethylene-4-diphenylmethylene-3-disubstitutedmethylene-tetrahydrofuran-2-one compounds have been prepared and structurally characterised. The compounds are obtained as yellow or dark red crystals, or, in one case, both. In two cases where yellow crystals were obtained, we found that crushing the crystals gave a deep red powder. Structure determinations, including those of the one compound which gave both coloured forms, depending on crystallisation conditions, showed that the yellow crystals contained molecules in which the structure comprised a folded conformation at the diphenylmethylene site, whilst the red crystals contained molecules in a twisted conformation at this site. Lattice energy and molecular conformation energies were calculated for all molecules, and showed that the conformational energy of the molecule in structure **IIIa** (yellow) is marginally higher, and the conformation thus less stable, than that of the molecule in structure **IIIb** (red). However, the van der Waals energy for crystal structure **IIIa**, is slightly stronger than that of structure **IIIb** – which may be viewed as a hint of a metastable packing preference for **IIIa**, overcome by the contribution of a more stabilising Coulomb energy to the overall more favourable lattice energy of structure **IIIb**.

**Conclusions:**

Our studies have shown that the crystal colour is correlated with one of two molecular conformations which are different in energy, but that the less stable conformation can be stabilised by its host crystal lattice.

**Graphical abstract:**
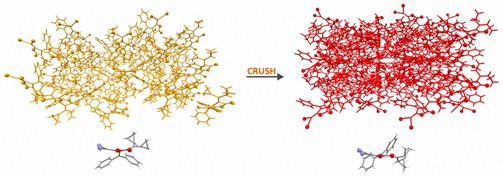
Graphical representation of the structural and colour change in the tribochromic compound (III).

**Electronic supplementary material:**

The online version of this article (doi:10.1186/s13065-014-0070-3) contains supplementary material, which is available to authorized users.

## Background

**Scheme 1:**
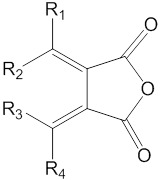
**The general formula of a fulgide.**

As part of a study of some fulgides (Scheme 1) with potential photochromic properties [1], a number of 5-dicyanomethylene-4-diphenylmethylene-3-disubstitutedmethylene-tetrahydrofuran-2-one compounds have been prepared and structurally characterised.

**Scheme 2:**
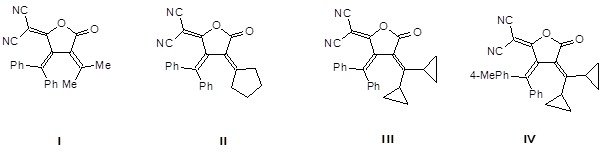
**The formulae of compounds I to IV.**

In this paper, we report on the preparations and structures of the modified fulgide compounds shown in Scheme 2, in which a dicyanomethylene group has replaced one of the carbonyl groups.

One compound, identified as **I**, was obtained as yellow crystals on crystallization from a deep red solution of 3:7 ethyl acetate and petroleum. Since the yellow form gave deep red solutions when re-dissolved, a photochromic change in solution on exposure to ultraviolet light could not be established. With a view to determine whether the yellow form was photochromic in the solid state, a crystal was crushed on a filter paper with the intention then of irradiating the expected yellow powder with ultraviolet light. By simply crushing the crystal, a dark red powder was produced which did not revert to the yellow form unless the red powder was re-crystallised from 3:7 ethyl acetate and petroleum. This seemingly irreversible solid state process of colour change by rubbing, grinding or fracturing the crystals, was termed by us as “tribochromism” from the Greek ‘tribos’ - rubbing and ‘chroma’ - colour. In order to explore this phenomenon further, additional compounds were synthesised with different substituents at the isopropylidene site, (see Scheme 2), but these exhibited variable properties. Thus, when the isopropylidene was replaced by a cyclopentylidene, the product **II** was obtained directly as deep red crystals. No yellow crystals could be obtained for this compound. A product which could be obtained as both yellow and red crystals was eventually obtained, when the methyl groups of compound **I** were replaced by cyclopropyl groups, giving compound **III**. In order to explore the relationship between structure and colour, we set out to determine the crystal structures of key products. These studies showed that the yellow (**IIIa**) and red (**IIIb**) forms from the solution of compound **III** are formally polymorphs of **III**, in which the molecules have different conformations. The structure of a fourth compound, **IV**, a very close analogue of **III**, but which gave only yellow crystals, was also determined, to check its molecular conformation, and also with a view to see how the slight difference in molecular structure would affect the crystal packing. The syntheses and structure determinations of **IIIa** and **IIIb** have been briefly described in a previous, short communication [2]. For this current paper, we have now had the opportunity to examine the relationship between the structures using computational chemistry techniques to ascertain the electronic structures of the molecules in the two forms, **a** and **b**, of compound **III**, and calculate lattice and related energies of these and the other, related crystal structures.

## Experimental

### a). Syntheses and crystallizations

All solvents were carefully dried before use. Petroleum refers to the fraction b.p. 60-80°C. The preparations of the first stage succinic anhydrides, and all characterisation data are described in the supplementary information, Additional file 1.

#### Compound I. 5-dicyanomethylene-4-diphenylmethylene-3-isopropylidenetetrahydrofuran-2-one

Diethylamine (0.48 g, 6.6 mmoles) was added to a mixture of diphenylmethylene(isopropylidene) succinic anhydride (1 g, 3.3 mmoles) and malononitrile (0.22 g, 3.3 mmoles) in tetrahydrofuran (15 ml) at 0°C and then stirred at room temperature (20 h). Solvent was removed and the residue treated with ether (5 ml). A colourless salt separated which was cyclised by stirring with acetyl chloride (1 ml) in dichloromethane (1 ml) for 6 hours. The residue was purified by chromatography on a column of silica gel (100 g) using a 3:7 mixture of ethyl acetate and petroleum as eluant. The dicyanomethylene compound was obtained as yellow crystals on evaporation of the deep red solution, m.p. 182-183°C (0.64 g, 55%).

#### Compound II. 5-dicyanomethylene-4-diphenylmethylene-3-cyclopentanylidentetrahydrofuran-2-one

Diethylamine (0.67 g, 9.2 mmoles) was added to a mixture of cyclopentanylidene (diphenylmethylene)succinic anhydride (1.5 g, 4.5 mmoles) and malononitrile (0.3 g, 4.5 mmoles) in tetrahydrofuran (15 ml) at 0°C and then stirred at room temperature (20 h). Solvent was removed and the residue treated with ether (5 ml). A colourless salt separated which was cyclised by stirring with acetyl chloride (1 ml) in dichloromethane (1 ml) for 6 hours. The residue was purified by chromatography on a column of silica gel (100 g) using a 3:7 mixture of ethyl acetate and petroleum as eluant. The dicyanomethylene compound was obtained as dark red crystals on partial evaporation of the solvent, m.p. 162-164°C (0.64 g, 55%) and was recrystallised from dichloromethane and petroleum.

#### Compound IIIa,b. 5-dicyanomethylene-4-diphenylmethylene-3-dicyclopropylmethylenetetrahydrofuran-2-one

Diethylamine (0.53 g, 7 mmoles) was added to a mixture of diphenylmethylene (isopropylidene) succinic anhydride (1.26 g, 3.5 moles) and malononitrile (0.24 g, 3.6 mmoles) in tetrahydrofuran (10 ml) at 0°C and then stirred at room temperature (12 h). Solvent was removed. On treatment of the residue with ether (5 ml), a colourless salt separated, which was cyclised as described above. The residue was purified by chromatography on a column of silica gel (100 g) using a 3:7 mixture of ethyl acetate and petroleum as eluant. On slow evaporation of the deep red eluant, yellow crystals of form **IIIa** of the dicyanomethylene compound (0.3 g, 21% yield) m.p. 183-184°C were initially obtained.

Further evaporation of the concentrated deep red solution gave dark red crystals of form **IIIb** of the same dicyanomethylene compound (0.1 g 7% yield) m.p. 188-189°C.

#### Compound IV(c,d). E- and Z- 5-dicyanomethylene-3-dicyclopropylmethylene-4-[4-methylphenyl)(phenyl)methylene]tetrahydrofuran-2-one

Diethylamine (0.59 g, 8.1 moles) was added to a mixture of 1:1 E: Z mixture of 4-methylphenyl (phenyl)methylene(dicyclopropylidene)succinic anhydride (1.50 g, 4.05 mmoles) and malononitrile (0.26 g, 4.05 mmoles) in tetrahydrofuran (10 ml) at 0°C and then stirred at room temperature (20 h). The solvent was removed, the residue dissolved in DCM (10.ml) and treated with acetyl chloride (10 ml) to give the dicyanomethylene compound as yellow crystals only. (M.p. 183–185°C). The single crystal selected for X-ray crystallography turned out to be the Z-isomer, **IV(d)**.

### b). X-ray crystallography

Data for the five crystals studied were recorded using a FAST TV Area Detector diffractometer, following procedures described in reference [2]. The crystal quality of compound **IIIa**, which was obtained in limited amounts, was poor, and this is reflected in the resulting quality of the structure. However, the reliability of the basic structural characterisation is without doubt. Crystallographic data for the five forms identified are listed in Table 1. CIF files for the three new structures have been deposited at the CCDC. The relevant Depcodes, along with the Refcodes for the two published structures are listed in Table 1. Cif files for compounds I-1V are provided as Additional files 2, 3, 4, 5, 6.

**Table 1:** **Selected crystal structure data**

Compound	I	II	IIIa	IIIb	IV
Formula	C_23_H_16_N_2_O_2_	C_25_H_18_N_2_O_2_	C_27_H_20_N_2_O_2_	C_27_H_20_N_2_O_2_	C_28_H_22_N_2_O_2_
F. Wt.	352.38	378.41	404.45	404.45	418.48
Space group	P2_1_	P2_1_/n	P2_1_/c	C2/c	P2_1_/c
a (Å)	10.352(1)	14.384	10.07(3)	20.349(2)	8.610(4)
b (Å)	7.212(1)	8.214	23.91(5)	10.095(2)	12.780(5)
c (Å)	13.320(1)	17.365	9.50(5)	21.338(2)	20.458(8)
α (deg)	90	90	90	90	90
β(deg)	110.33(1)	105.53(1)	110.4(2)	99.06(2)	101.72(3)
γ (deg)	90	90	90	90	90
Z/Z’	2/1	4/1	4/1	8/1	4/1
V (Å^3^)	932.49	1976.7(2)	2144(13)	4328.6(10)	2204.19(16)
R/Rw	0.043/0.126	0.041/0.114	0.089/0.207	0.062/0.159	0.043/0.084
CCDC Dep/**Ref** code	1009944	1009945	**DOQNAY01**	**DOQNAY**	1009946

### c). Computational studies

#### i) Lattice energy calculations

Solid state calculations of the lattice energies and the crystal structures of **I**, **II**, **IIIa**, **IIIb** and **IV** were carried out using the GRACE package [3]. GRACE provides an efficient algorithm for optimising the co-ordinates and lattice parameters of molecular crystals. Lattices energies and gradients are provided by a solid state density functional theory method with corrections for dispersive interactions (the DFT-D method) [4]. GRACE uses the VASP [5] program with the PW91 density functional [6] to calculate the lattice energy and its gradients. The dispersive correction is provided by a damped molecular mechanical potential. Starting with the experimental crystal structures, the unit cells and molecular geometries were fully optimised within the constraints of the experimental space group symmetries.

The DFT-D lattice energy is defined by the energy of the process of going from molecules in the ‘gas phase’ to the crystal, with the lowest energy conformer of each molecule taken as the reference energy. It is possible to break down the contributions to the lattice energy into terms arising from conformational, conformational deformation, van der Waals and Coulombic components. In the following description of the solid state calculations a ‘molecular’ or ‘gas phase’ calculation refers to a DFT-D calculation of a single molecule in a unit cell which is large enough that a molecule does not interact with images of itself. Typically unit cells with dimensions of over 23 Å were needed to ensure convergence of the molecular calculations. In these calculations the molecular structure was optimised but the unit cell and the orientation of the molecule in the unit cell was held fixed. A ‘crystal calculation’ refers to a DFT-D optimisation of the crystal structure in which the crystal structure, comprising the molecular geometry, the molecular orientation and the unit cell dimensions were fully optimised. In order to estimate the Coulombic contribution to the DFT-D lattice energy, atomic charges which reproduce the molecular electrostatic potential associated with the optimised molecule were calculated using GRACE. These point charges were then used to calculate the change in Coulomb energy on going from the molecule in the ‘gas-phase’ to the solid state. The van der Waals' contribution was estimated from the difference in the molecular and solid state dispersive energy corrections used by the DFT-D method. The conformational contribution arises from the difference in energies between molecularly optimised conformations of the same molecule, whilst the conformational deformation energy arises from the change in energy of a conformation on moving from the gas phase to the solid state. The conformational deformation energy was calculated from molecular calculations of the energy difference between a molecule optimised in the solid state and in the gas phase. The sum of the conformational deformation and conformational energy terms represents the increase in internal energy of the molecule due to its presence in the crystal. It follows that the ‘deformation’ term and the conformational energy term are always positive energies. The remaining contributions to lattice energy, including terms arising from polarisation and induction, are calculated from the differences between the total lattice energies and the sum of all the other contributions; conformational, conformational deformation, van der Waals and Coulomb.

#### (ii) Molecular calculations

Molecular calculations were carried out with the ORCA package [7]. Geometry optimisations were performed starting with molecular structures extracted from the experimental crystal structures. The structures were optimised using the TZVPP basis [8] with the BLYP density functional, including a dispersive correction [9]. The basis set consists of 5 s, 3p, 2d and 1f functions on C, N and O, and 3 s, 2p and 1d functions on H. Calculations of the electronic excitation energies for the lowest excited singlet states for each molecule were performed at the resulting optimised geometries. The time dependent density functional method using the B2PLYP functional [10] was used to calculate the excited state energies and wavefunctions. The B2PLYP functional is a double hybrid functional which is a combination of the generalized gradient approximations for the correlation and exchange, the Hartree-Fock exchange and a second order correlation term which is included by perturbation theory [11]. It has been shown to provide reasonable predictions of the excited state energies of organic molecules [11],[12]. A TZVP basis was used [8] for the calculation of the excited state energies. The basis set consists of 5 s, 3p and 1d functions on C, N and O, and 3 s and 1p functions on H and is the same as the basis set used in the study of Goerigk et al. [12]. To estimate the effect of the crystal environment on the absorption spectrum, molecular geometry optimisations and calculations of the first excite states were repeated using the COSMO solvation model [13] using an effective medium with a refractive index of 75 and a dielectric constant of 3.0 which is representative of an organic molecular crystalline environment [14].

Excited state calculations at the geometry of the optimised ground state give vertical excitation energies. In order to estimate the adiabatic excitation energies, which are more appropriate for comparison with experiment, calculations of the optimised ground and excited states were performed using the TZVP basis with the BLYP functional. For these calculations no dispersive correction was employed. For each molecule an adiabatic correction was determined by calculating the energy change on optimising the first excited state starting at the optimised ground state geometry.

### d) Molecular packing studies

**Figure 1:**
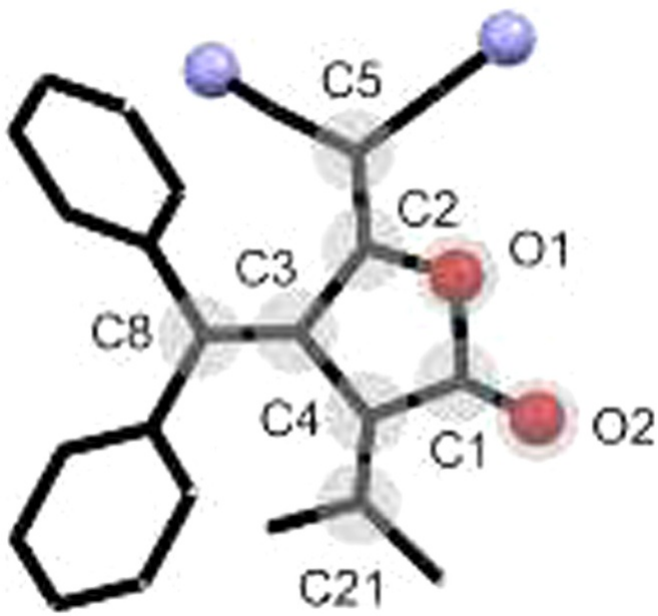
**Atoms used (shaded grey) to define the COSP for the XPac calculation.**

A molecular packing analysis was made on the five structures, using the XPac program [15]. The atoms defined in Figure 1 were used as the COSP (Corresponding Ordered Set of Points) [15].

## Results and discussion

### Crystal structures

**Figure 2:**
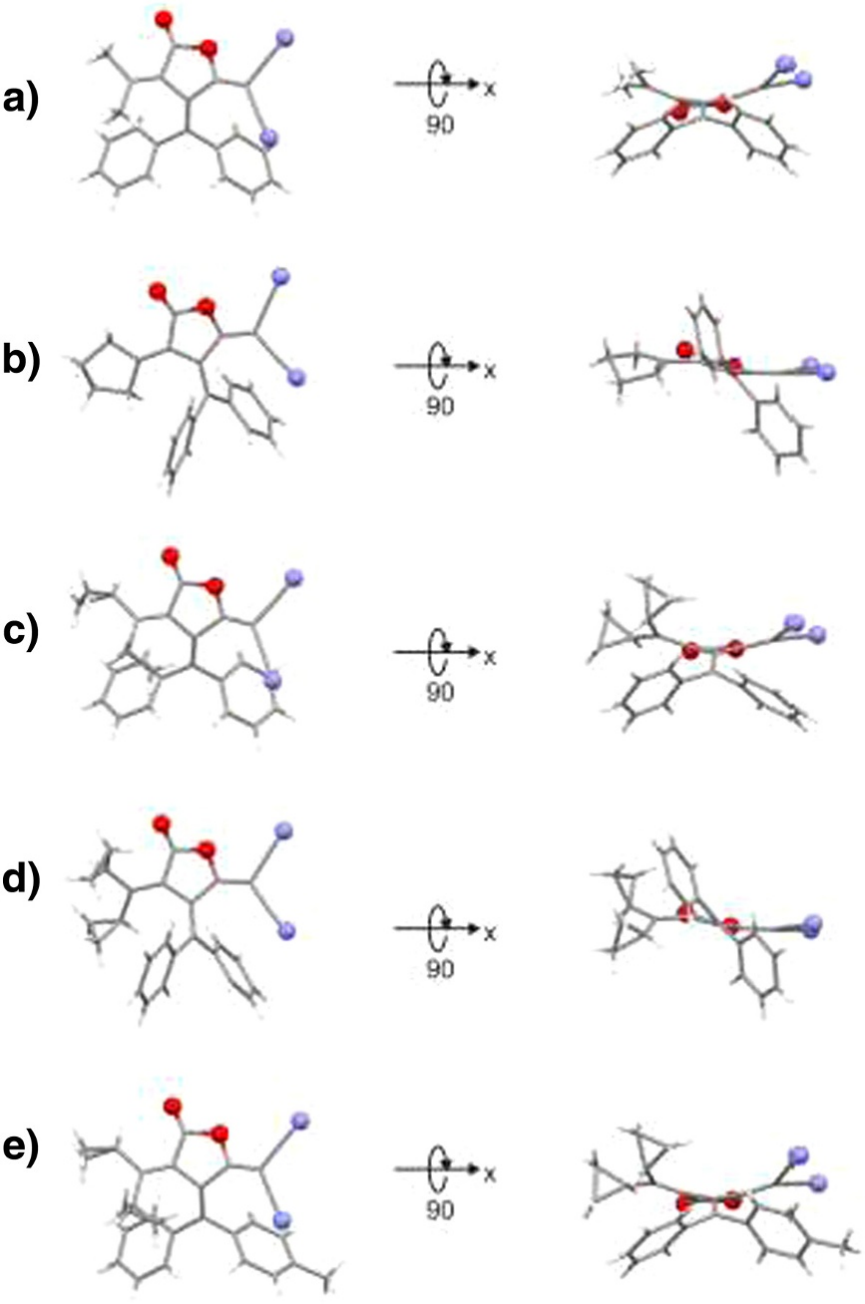
**Folded (a, c and e) and twisted (b and d) conformations viewed along perpendicular directions (left and right): a) I; b) II; c) IIIa; d) IIIb; e) IV.**

As mentioned in our earlier communication [16], the crystal structure determination of both **IIIa** and **IIIb**, the one example where we were able to obtain useable single crystals of both the yellow and red forms of the same compound, indicated that the molecules in the two forms showed dramatic differences in conformation. In the case of the yellow form the molecule has a folded conformation which is characterised mainly by an envelope fold of the tetrahydrofuran-2-one ring, and a very slight pyramidalization at the carbons at each end of the diphenylmethylene double bond; in the case of the red crystals, the molecule is twisted about the equivalent methylene component. Here, the main deformation is a rotation of the two groups at the ends of the diphenylmethylene bond. The structures determined for compound **I**, **II** and **IV** revealed that the two conformations are repeated, and are consistent with the crystal colour. Thus the molecules in the structure of the yellow crystals of **I** and **IV** are folded, and in the structure of the red crystals of **II** are twisted. Figures 2a-e, demonstrate the reality of these very different molecular conformations. The Figures are drawn using the Mercury software package [17].

**Figure 3:**
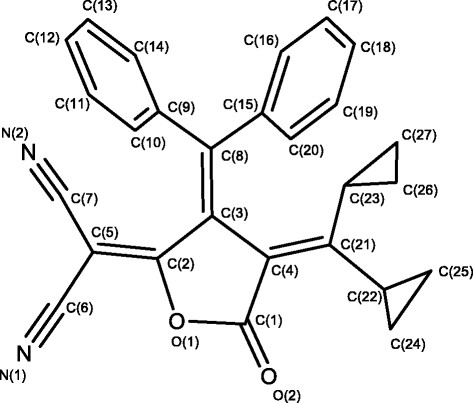
**The molecular structure of compound IIIb, showing the atom numbering scheme.** All heavy atoms up to C23 have the same numbering in all five structures. Hydrogen atoms are omitted for clarity.

We can view the two forms of **III** as special examples of conformational and colour polymorphism, both of which have been long known and intermittently revisited [18]–[21], and frequently related. Reference [20] provides an excellent summary of much of the history and previous work on colour polymorphism, and reference [21] provides a detailed analysis of conformational polymorphism. As mentioned in reference [20], colour polymorphism is generally found to be related to differences in molecular conformation in the crystals, usually associated with conformational changes about a formally single bond coupled to a double bond. The generally small changes in colour – e.g. yellow/orange were explained in terms of small changes in the wavelengths. The dramatic difference in the two structures found in this work, which relates to significant, “twist/fold” conformational differences about a double bond, clearly merit detailed assessment. Figure 3 shows the atom numbering scheme of the molecule of compound **III**. Equivalent atoms, especially those which define important groups, relating to the conformational differences have the same numbering in all molecules. Figures of the four molecules with specific atom numbering are given in the ESI.

### Molecular packing analysis

**Figure 4:**
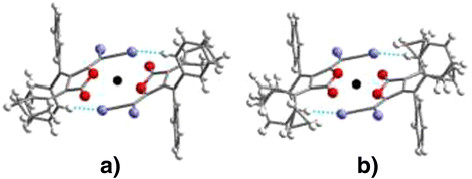
**The weak C-H…NC hydrogen-bonded dimers in structure II (a) and structure IIIb (b).** The central black dots indicate centres of inversion.

The Xpac study revealed only one similarity between the five crystal structures, a zero dimensional similarity based on an analogous orientation of the chosen COSP in structures **II** and **IIIb**. This similarity is a centrosymmetric arrangement of this common molecular core, in which one of the cyano-nitrogen atoms is weakly H-bonded to a phenyl hydrogen, as can be seen in Figure 4. The N…H distance is 2.75 Å in II and 2.65 Å in **IIIa**. Since this feature occurs only in these two forms, both containing the twisted molecular conformation, it may contribute to helping stabilise this conformation. Otherwise, the molecular packings are quite different in the five structures.

### Computational studies

#### (i) Crystal structure calculations

As can be seen from Table 2 the optimised unit cells are in good agreement with the experimental unit cells. The largest deviation (−3.1%) is associated with the *a* parameter of the unit cell of **I**. The agreement between the experimental and optimised unit cells instils confidence that the DFT-D method is providing a good description of the crystals, confirming our previous experience with this method [22]–[27].

**Table 2:** **Comparison of DFT-D optimised and experimental unit cells**

		Unit cell parameters				RMSD*
**Crystal**	**Method**	***a*** **(Å)**	***b*** **(Å)**	***c*** **(Å)**	***β*** **(°)**	**(Å)**
**I**	Exptl.	10.352	7.212	13.320	110.3	
	DFT-D	10.027	7.169	13.419	110.4	0.257
	Deviation (%)	−3.1	−0.6	0.7	0.0	
**II**	Exptl.	14.384	8.214	17.365	105.5	
	DFT-D	14.378	8.282	17.373	105.4	0.071
	Deviation (%)	0.0	0.8	0.0	−0.2	
**IIIa**	Exptl.	10.070	23.910	9.500	110.4	
	DFT-D	10.160	23.357	9.458	109.6	0.190
	Deviation (%)	0.9	−2.3	−0.4	−0.7	
**IIIb**	Exptl.	20.349	10.095	21.338	99.1	
	DFT-D	20.126	10.081	21.242	98.1	0.163
	Deviation (%)	−1.1	−0.1	−0.5	−1.0	
**IV**	Exptl.	8.610	12.780	20.458	101.7	
	DFT-D	8.626	12.709	20.344	102.1	0.104
	Deviation (%)	0.2	−0.6	−0.6	0.4	

*The RMSD is the root mean squared deviation in the atomic positions of a matching cluster of 15 molecules in the experimental and optimised crystals according to the crystal similarity tool in the Mercury software program^16^.

The final lattice energy of each crystal structure is shown in Table 3 along with a breakdown of the energy into the various contributions

**Table 3:** **Breakdown of contributions to the total DFT-D lattice energies**

Crystal	Conformational energy (kcal mol ^−1^)	Conformational deformation energy (kcal mol ^−1^)	van der Waals energy (kcal mol ^−1^)	Coulomb energy (kcal mol ^−1^)	Remaining energy (kcal mol ^−1^)	Lattice energy (kcal mol ^−1^)
**I**	0.0	0.7	−33.2	−9.7	2.9	−39.4
**II**	0.0	0.2	−34.6	−9.5	1.5	−42.5
**IIIa**	1.6	1.0	−36.2	−9.3	2.1	−40.8
**IIIb**	0.0	1.0	−34.9	−11.4	0.0	−45.3
**IV**	0.0	1.4	−38.4	−8.7	1.8	−43.9

In the case of the two polymorphs of **III**, form **IIIb** is calculated to be 4.5 kcal mol^−1^ more stable than **IIIa**. The origin of the stability difference is complex. Although the molecular conformation found in **IIIa** is 1.6 kcal mol^−1^ higher in energy than that in **IIIb**, this is compensated for, in part, by the van der Waals' contribution which favours **IIIa** by 1.2 kcal mol^−1^. The stability of **IIIb** relative to **IIIa** seems to come from an increased Coulombic contribution (2.1 kcal mol^−1^) along with a further 2.1 kcal mol^−1^ related to other contributions such as induction effects.

For all crystals the conformational deformation contribution to the lattice energy is quite small (less than 1.4 kcal mol^−1^). The van der Waals' contribution is the largest contribution (between 77 and 89% of the total lattice energy) and generally gets larger as the molecules get larger. The Coulombic contribution varies between 20 and 25% of the total and the contribution whose origin has not been attributed varies between 4 and 7% of the total.

The main indications which are provided by these results relate essentially to the polymorphism of compound III, and can be summarized as follows:

i) The conformational energy of the molecule in structure **IIIa** is marginally higher, and the conformation thus less stable, than that of the molecule in structure **IIIb**
ii) The van der Waals energy for crystal structure **IIIa**, is slightly stronger than that of structure **IIIb** – which may be viewed as a hint of a metastable packing preference for **IIIa**, overcome, however, by the contribution of a more stabilising Coulomb energy to the overall more favourable lattice energy of structure **IIIb**.

The break-down final lattice energies proposed here is not unique. A method based on gas phase optimisation of a conformer starting from its experimental structure as found in the crystal has been used by Bernstein and Cruz-Cabeza [21]. The Pixel method [28], which uses the gas phase electron density of the molecule to calculate the interaction energies in the experimental or calculated crystal structure has also been used.

#### (ii) Molecular calculations

Table 4 gives the optimised molecular energies and reports the vertical excitation energies, with and without consideration of the environment. The molecular calculations predict that the conformation of the molecule in polymorph **IIIa** is less stable than that in polymorph **IIIb**, with an energy difference of 0.8 kcal mol^−1^. This compares with a value of 1.6 kcal mol^−1^ found using VASP with the PW91 functional (see Table 3). The agreement between the methods is good, although there will be errors in both calculations due to the choice of density functional and basis set. In the case of VASP an additional uncertainty is provided by the choice of pseudopotential. The molecular calculations could be improved by the explicit consideration of electron correlation effects through the use of couple cluster calculations. However, this was not warranted given the agreement between the two theoretical methods.

**Table 4:** **Molecular energies, HUMO-LUMO gap and the predicted wavelengths associated with the first excited states**

Molecule	Absolute energies ^a^(Hartree)	Relative energies ^a^kcal/mol	HOMO LUMO ^a^Gap (eV)	Gas phase 1 ^st^Excited state ^b^(eV)	COSMO 1 ^st^Excited state ^b^(eV)	Adiabatic Correction ^c^(eV)
**I**	−1146.053748468		2.58	3.71	3.38	0.53
**II**	−1223.470314612		1.55	2.61	2.41	-^d^
**IIIa**	−1300.807815239	0.8	2.49	3.63	3.16	0.78
**IIIb**	−1300.809021877	0.0	1.45	2.46	2.23	-^d^
**IV**	−1340.117586577		2.40	3.47	3.05	0.73

^a^The energies are based on calculations using the BLYP density functional with a dispersion correction. ^b^The excited state energies are calculated using time dependent DFT with the B2PLYP functional at the ground state optimised geometry. ^c^The adiabatic correction is the excited state energy change on going from the optimised geometry of the excited state to that of the ground state. ^d^The geometry optimisation failed to converge.

For all molecules the wavefunction of the first excited state is dominated by a single electronic configuration, which arises from the transfer of an electron from the HOMO to the LUMO. It is not surprising therefore that there is a good correlation between the calculated HOMO-LUMO energy gap and the energy of the first excited state. The partial inclusion of the influence of the crystal environment through the COSMO model introduces a reduction in the calculated energy of the first excited stated by between 0.20 and 0.47 eV. Attempts were made to calculate the optimised geometries of the excited states in order to estimate the adiabatic excitation energies. In the cases of molecules **I**, **IIIa** and **IV**, optimised geometries were determined. For molecules **II** and **IIIb**, both of which have twisted conformations, geometry optimisations failed because large changes in the molecular geometries were causing excited states to cross during the optimisation processes.

#### (iii). Molecular geometry comparisons

We have a number of ways in which we can identify and discuss the detailed structural relationships between these compounds. As a first approach we consider the bond lengths and bond angles in the molecules, which already reveal some interesting features, and then consider the variations in the torsion angles at key positions. These data are shown in Table 5.

- Bond Lengths. In the 5-membered ring, the O(1)-C(2) bond is consistently shorter than the C(1)-O(1) bond, indicating a strengthened interaction between O(1) and the dicyanomethylene carbon C(2). This is likely to be a feature of the fundamental electronic structure of the trimethylene substituted tetrahydrofuran-2-one, and particularly the dicyanomethylene group, since this bonding distribution is also found in the structurally related molecules LORMAH [29], MUZBOY [30] and YETBEE [31], each of which contains this component. The calculated, minimised structures **IIIa**(C) and **IIIb**(C) both capture this result also.
  A second feature, again captured by the calculations, is the small, but consistent lengthening – 0.03-0.04 Å, of the diphenylmethylene double bond, C(3) = C(8), in the twisted, red forms, compared with the folded forms.
- Bond Angles. Here we examine the angles involving the tetrahydrofuran-2-one ring and its organic substituents. A first, simple calculation of the sum of the internal angles of the ring shows that the twisted structures of **II** and **IIIb** retain quite planar rings (sum of internal angles are 539.5° and 539.6°, whilst the folded structures have rings with internal angles summing to 533.1° and 535.2°, reflecting the formal reduction of ring angles at a fold. An additional point of interest is the comparison of the external angles at each of the methylene substituents, where distortions may occur due to differences in intramolecular interactions with neighbouring groups. Thus, we find that for the dicyanomethylene function, the O(1)-C(2) = C(5) and C(3)-C(2) = C(5) angles show a difference of 11-20°, with the larger angles on the side which clashes with the diphenylmethylene group. For this latter group itself, the angles differ only by 1-4°, whilst the methylene group at C(4) shows an intermediate distortion of 8-12°, again with the larger angle due to clashes with the diphenyl functions.
- Dihedral and Torsion Angles. Clearly, the critical parameters for the major distortions in the folded, yellow molecules are the angles of fold across C(2)…C(4), which have values of 23.7(3)^o^, 20.9(10)^o^, 23.7(2)^o^ for **I**, **IIIa** and **IV**, respectively. Whilst it logical to quantify the twist about the C(3) = C(8) bonds in **II** and **IIIb** by the dihedral angles between the two planes C(2),C(3),C(4) and C(9),C(8),C(15), which have values of 37.4(2)^o^ and 35.7(3)^o^ for structures **II** and **IIIb**, these do not give the full story, and it is interesting to compare the important torsion angles also. Values of these angles will indicate whether the distortions in the bond angles mentioned above are essentially in-plane or whether any unbalanced intramolecular repulsions again occur, which may “distort the distortions”. The torsion angles we are interested are the *cis* bonds at the ends of each of the carbon-carbon double bonds, typified by the pair O(1)-C(2) = C(5)-C(6) and C(3)-C(2) = C(5)-C(7). The relevant data is presented in the last section of Table 2, and shows that the differences vary from 0.5° to 17°. There is no marked difference related to the folded or twisted conformations, but, in general, the larger values are obtained for the structures containing the bis-cyclopropyl substituent, indicating the effect of the accommodation of the two bulky ligands in these molecules.

**Table 5:** **Selected, comparative molecular geometry parameters for the five structures; X denotes X-ray results, C the results from the computational study**

Parameter	I(X)	I(C)	II(X)	II(C)	IIIa(X)	IIIa(C)	IIIb(X)	IIIb(C)	IV(X)	IV(C)
C(1)-O(1)	1.433	1.449	1.413	1.428	1.39	1.457	1.415	1.437	1.432	1.455
C(1)-C(4)	1.463	1.488	1.474	1.483	1.47	1.480	1.464	1.487	1.479	1.480
O(1)-C(2)	1.364	1.372	1.370	1.376	1.33	1.369	1.362	1.370	1.365	1.369
C(2)-C(3)	1.474	1.475	1.457	1.457	1.51	1.473	1.447	1.458	1.480	1.473
C(3)-C(4)	1.497	1.494	1.467	1.475	1.49	1.488	1.464	1.472	1.476	1.488
C(2) = C(5)	1.350	1.376	1.367	1.393	1.32	1.376	1.379	1.393	1.363	1.377
C(3) = C(8)	1.348	1.370	1.389	1.408	1.36	1.371	1.397	1.409	1.363	1.373
C(4) = C(21)	1.344	1.363	1.357	1.371	1.37	1.376	1.360	1.390	1.362	1.376
O(1)-C(2) = C(5)	115.8	118.4	114.2	115.1	119.3	118.8	113.0	115.2	116.3	118.5
C(3)-C(2) = C(5)	134.8	132.0	134.1	133.4	130.5	131.5	136.1	133.5	134.9	131.7
O(1)-C(2)-C(3)	109.4	109.3	110.7	110.7	110.1	109.3	110.1	110.4	106.7	109.4
C(2)-C(3) = C(8)	126.7	125.9	125.3	125.9	128.0	103.5	126.5	105.5	125.0	126.3
C(4)-C(3) = C(8)	128.4	128.6	129.7	128.8	127.4	127.7	127.4	128.9	128.8	127.6
C(2)-C(3)-C(4)	102.8	103.2	105.0	105.3	100.7	103.5	105.7	105.5	103.7	103.6
C(3)-C(4) = C(21)	131.1	131.3	131.6	131.8	131.9	130.6	129.7	130.7	131.6	130.9
C(1)-C(4) = C(21)	122.7	123.3	119.4	119.4	121.7	124.2	121.6	120.1	123.1	123.9
C(1)-C(4)-C(3)	105.2	104.7	106.9	107.0	106.2	104.9	106.7	107.0	106.1	105.0
C(4)-C(1)-O(1)	107.0	106.3	107.4	106.9	107.0	106.3	107.0	106.3	107.1	106.3
C(1)-O(1)-C(2)	109.6	109.2	109.5	109.6	111.2	109.0	110.1	109.9	109.4	109.2
O(1)-C(2) = C(5)-C(6)	−0.9	−5.9	−2.1	−9.7	−6.2	−6.3	−2.3	−9.6	−6.8	−6.1
C(3)-C(2) = C(5)-C(7)	−4.3	−2.4	3.0	−3.4	−8.7	−2.1	1.8	−3.4	−10.4	−1.8
C(2)-C(3) = C(8)-C(9)	6.3	−2.9	−39.0	−40.3	6.3	−3.3	−40.3	−39.9	−10.6	−3.9
C(4)-C(3) = C(8)-C(15)	−3.7	−11.3	−35.7	−38.3	−11.0	−12.3	−30.4	−33.3	−2.2	−13.2
C(3)-C(4) = C(21)-C(23)	2.3	4.7	0.9	0.9	19.4	16.8	−12.0	−15.2	9.0	17.9
C(1)-C(4) = C(21)-C(22)	14.0	14.0	−12.0	−12.0	23.3	22.5	−29.3	−31.4	16.3	23.1

ESD’s for Experimental data are provided in the Cif files in the ESI. Distances are in Ångstroms and angles in degrees.

## Conclusions

The theoretical calculations of the parameters defining the crystal and molecular structures, map very well with those found experimentally. The molecular and lattice energy calculations indicate that the folded yellow conformation of compound **III** has a higher molecular energy than the twisted red conformation, but that the crystal packing energy is lower, and supports the less stable conformation in structure **IIIb** and presumably in structure **II**, for which no red crystals could be obtained. However, we presume that the twisted form is predominant in all solutions, which are red. These results will, no doubt, be of particular interest to colleagues engaged in structure prediction calculations.

## Additional files

## Electronic supplementary material

Additional file 1: **Electronic supplementary information (ESI).** A. Molecular structures of compounds **I-IV** with atom numbering (Hydrogen atoms omitted for clarity). B. Preparation of starting materials. (DOCX 101 KB)

Additional file 2: Cif file for structure I. (ZIP 5 KB)

Additional file 3: Cif file for structure II. (ZIP 5 KB)

Additional file 4: Cif file for structure IIIa. (ZIP 5 KB)

Additional file 5: Cif file for structure IIIb. (ZIP 5 KB)

Additional file 6: Cif file for structure IV. (ZIP 5 KB)

Below are the links to the authors’ original submitted files for images.

Authors’ original file for figure 1

Authors’ original file for figure 2

Authors’ original file for figure 3

Authors’ original file for figure 4

Authors’ original file for figure 5
