# Supplementary material for: A mechanophysical phase transition provides a dramatic example of colour polymorphism: the tribochromism of a substituted tri(methylene)tetrahydrofuran-2-one
Source: Chem Cent J. 2014 Dec 16;8:70. doi: 10.1186/s13065-014-0070-3 (PMC4266767; doi:10.1186/s13065-014-0070-3)
Supplement: Supplementary file 1 — Additional file 1: Electronic supplementary information (ESI). A. Molecular structures of compounds I-IV with atom numbering (Hydrogen atoms omitted for clarity). B. Preparation of starting materials. (DOCX 101 KB) [file 13065_2014_70_MOESM1_ESM.docx]

Electronic supplementary information (ESI)

A. Molecular structures of compounds **I-IV** with atom numbering (Hydrogen atoms omitted for clarity)

|   I |   II |
| --- | --- |
|   III |   IV |

B. Preparation of starting materials.

Diethyl isopropylidenesuccinate

A mixture of diethyl succinate (174 g, 1 mole), acetone (71 g, 1.2 moles) and dry potassium t-butoxide (112 g, 1 mole) in toluene (500 ml) was stirred in a 2l r.b. flask for 12 hours. The reaction mixture was poured onto crushed ice (500 g), the aqueous layer separated, and the toluene layer washed with water (3 x 100 ml). The combined aqueous layer was carefully acidified (over acidification causes the half ester to re-dissolve) with conc. hydrochloric acid causing the half ester to separate as an oil, which was dissolved in ether and dried over anhydrous magnesium sulphate. The dried half ester (134 g) was dissolved in ethanol (200 ml) and thionyl chloride (119 g, 1 mole) added drop wise. The mixture was refluxed (6 h). Solvent was removed under reduced pressure, the residual oil dissolved in ether, washed with 5% sodium bicarbonate solution, dried over anhydrous magnesium sulphate and solvent removed. The crude diester (93 g, overall yield 43 %) was obtained as a pale yellow oil, which was used without further purification.

Dimethyldiphenylmethylenesuccinate

A mixture of diethyl succinate (50 g, 0.34 moles), benzophenone (70 g, 0.38 moles) and dry potassium t-butoxide (50 g, 0.45 moles) in toluene (250 ml) was stirred in a r.b. flask (1 l) for 12 hours. The reaction mixture was poured onto crushed ice (250 g), the aqueous layer separated, and the toluene layer washed with water (2 x 100 ml). The combined aqueous layer was carefully acidified with conc. hydrochloric acid, the half ester separated as an oil. The dried half ester (78 g) was dissolved in methanol (200 ml) and acetyl chloride (40 g, 5 moles) added drop wise. The mixture was refluxed (6 h). Solvent was removed under reduced pressure and the residual oil dissolved in ether, washed with 5% sodium bicarbonate solution, separated, dried over anhydrous magnesium sulphate and solvent removed. The diester (79.6 g. overall yield 75%) was obtained as viscous yellow oil which crystallized on standing (m.p. 78-80^o^C).

Diphenylmethylene(isopropylidene)succinic anhydride. A mixture of diethyl isopropylidenesuccinate (30 g, 0.14 moles), benzophenone (26 g, 0.14 moles) and dry potassium t-butoxide (23.5 g, 0.2 moles) in toluene (200 ml) was stirred in a r.b. flask for 5 hours. Work-up, as above, gave the crystalline half ester (44 g) which was hydrolysed to the diacid by boiling (2 h) with 10% w/v ethanolic KOH (150 ml). Ethanol was removed under reduced pressure. The residual potassium salt was dissolved in water and acidified carefully with c. hydrochloric acid. The crude diacid was cyclised by boiling (1 h) with acetyl chloride (100 ml) in dichloromethane (100 ml). The fulgide was obtained as yellow crystals (15 g, 35% yield), m.p.167-168^o^C from ethanol.

E- and Z-dicyclopropylmethylene(4-methylphenyl(phenyl))methylenesuccinic anhydride

A mixture of diethyl dicyclopropylmethylenesuccinic acid (9 g, 0.042 moles), 4-methylbenzophenone (8.23 g, 0.0.042 moles) and dry potassium t-butoxide (5.65 g, 0.05 moles) in THF (70 ml) was stirred in a r.b. flask for 6 hours. Work-up gave the half ester as a brown gum (32.5 g) which was hydrolysed and cyclised to give the fulgides, which were purified by recrystallization from methanol as a 1:1 mixture of E- and Z-fulgides as yellow crystals (5.4 g, 35% yield).

Dicylopropylmethylene(diphenylmethylene)succinic anhydride

A mixture of equimolar proportions of diethyl dicyclopropylmethylenesuccinate [prepared using dicylopropyl ketone in place of acetone, using the procedure described above] (20 g, 0.84 moles), benzophenone (15.3 g, 0.084 moles) and dry potassium t-butoxide (10 g, 0.084 moles) in toluene (150 ml) was stirred in a r.b. flask for 24 hours. Work-up gave the half ester (10.73 g) which was hydrolysed and cyclised as described above. The fulgide was obtained as yellow crystals (4.2 g 14%) overall, m.p. 186-188^o^C from ether.

Cyclopentanylidene(diphenylmethylene)succinic anhydride

A mixture of dimethyl diphenylmethylenesuccinate (25 g, 0.8 moles), cyclopentanone (7 g, 0.08 moles) and dry potassium t-butoxide (15 g, 0.13 moles) in toluene (150 ml) was stirred in a r.b. flask for 30 hours. Work-up, as above, gave the half ester as a reddish gum (27.2 g) which was hydrolysed to the diacid (21.8 g). The fulgide was obtained as yellow crystals (6 g, 23% yield), m.p. 169-171^o^C from ethanol.
