# Supplementary figures and images for: A mechanophysical phase transition provides a dramatic example of colour polymorphism: the tribochromism of a substituted tri(methylene)tetrahydrofuran-2-one
Source: Chem Cent J. 2014 Dec 16;8:70. doi: 10.1186/s13065-014-0070-3 (PMC4266767; doi:10.1186/s13065-014-0070-3)

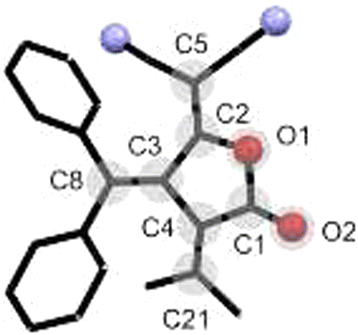

Supplement: Supplementary file 7 — Authors’ original file for figure 1 [file 13065_2014_70_MOESM7_ESM.gif]

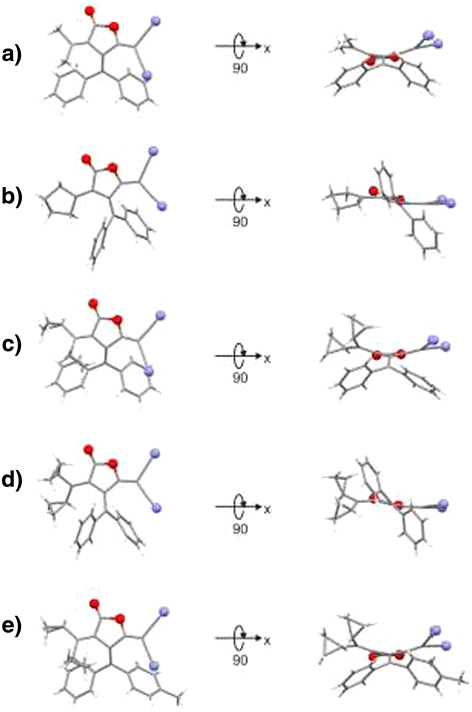

Supplement: Supplementary file 8 — Authors’ original file for figure 2 [file 13065_2014_70_MOESM8_ESM.gif]

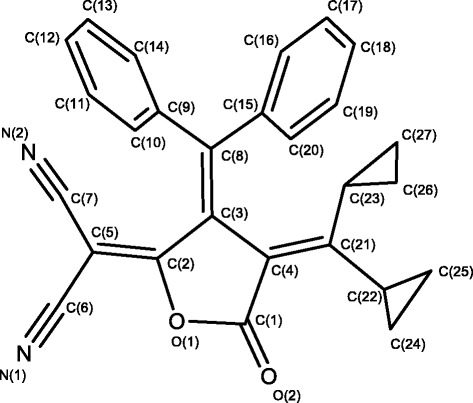

Supplement: Supplementary file 9 — Authors’ original file for figure 3 [file 13065_2014_70_MOESM9_ESM.gif]

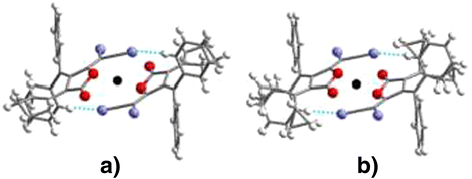

Supplement: Supplementary file 10 — Authors’ original file for figure 4 [file 13065_2014_70_MOESM10_ESM.gif]
